# Occupation and Risk of Non-Hodgkin Lymphoma and Its Subtypes: A Pooled Analysis from the InterLymph Consortium

**DOI:** 10.1289/ehp.1409294

**Published:** 2015-09-04

**Authors:** Andrea ‘t Mannetje, Anneclaire J. De Roos, Paolo Boffetta, Roel Vermeulen, Geza Benke, Lin Fritschi, Paul Brennan, Lenka Foretova, Marc Maynadié, Nikolaus Becker, Alexandra Nieters, Anthony Staines, Marcello Campagna, Brian Chiu, Jacqueline Clavel, Silvia de Sanjose, Patricia Hartge, Elizabeth A. Holly, Paige Bracci, Martha S. Linet, Alain Monnereau, Laurent Orsi, Mark P. Purdue, Nathaniel Rothman, Qing Lan, Eleanor Kane, Adele Seniori Costantini, Lucia Miligi, John J. Spinelli, Tongzhang Zheng, Pierluigi Cocco, Anne Kricker

**Affiliations:** 1 Centre for Public Health Research, Massey University, Wellington Campus, Wellington, New Zealand; 2 Department of Environmental and Occupational Health, Drexel University School of Public Health, Philadelphia, Pennsylvania, USA; 3 Tisch Cancer Institute, Icahn School of Medicine at Mount Sinai, New York, New York, USA; 4 Division of Environmental Epidemiology, Institute for Risk Assessment Sciences, Utrecht University, Utrecht, the Netherlands; 5 Department of Epidemiology and Preventive Medicine, Monash University, Melbourne, Australia; 6 School of Public Health, Curtin University, Bentley, Western Australia, Australia; 7 International Agency for Research on Cancer, Lyon, France; 8 Department of Cancer Epidemiology and Genetics, Masaryk Memorial Cancer Institute, Brno, Czech Republic; 9 Biological Hematology Unit, University of Burgundy, Dijon, France; 10 Division of Cancer Epidemiology, German Cancer Research Center Heidelberg, Heidelberg, Germany; 11 Center for Chronic Immunodeficiency, University Medical Center Freiburg, Freiburg, Germany; 12 School of Nursing and Human Sciences, Dublin City University, Dublin, Ireland; 13 Department of Public Health, Clinical and Molecular Medicine, University of Cagliari, Cagliari, Italy; 14 Department of Public Health Sciences, University of Chicago, Chicago, USA; 15 Epidemiology and Biostatistics Sorbonne Paris Cité Center (CRESS), Institut national de la santé et de la recherche médicale (INSERM), Villejuif, France; 16 French National Registry of Childhood Hematological Malignancies (NRCH), Villejuif, France; 17 Cancer Epidemiology Research Programme, Catalan Institute of Oncology–Spain Institut d’Investigació Biomèdica de Bellvitge (IDIBELL), Barcelona, Spain; 18 CIBER en Epidemiología y Salud Pública (CIBERESP), Barcelona, Spain; 19 Division of Cancer Epidemiology and Genetics, National Cancer Institute (NCI), National Institutes of Health (NIH), Department of Health and Human Services (DHHS), Bethesda, Maryland, USA; 20 Department of Epidemiology and Biostatistics, School of Medicine, University of California San Francisco, San Francisco, California, USA; 21 Radiation Epidemiology Branch, Division of Cancer Epidemiology and Genetics, NCI, NIH, DHHS, Bethesda, Maryland, USA; 22 Centre d’investigation clinique (CIC), INSERM, Bordeaux, France; 23 Registre des Hémopathies Malignes de la Gironde, Institut Bergonié, Bordeaux, France; 24 Occupational and Environmental Epidemiology Branch, Division of Cancer Epidemiology and Genetics, NCI, NIH, DHHS, Bethesda, Maryland, USA; 25 Epidemiology and Cancer Statistics Group, Department of Health Sciences, University of York, York, United Kingdon; 26 Environmental and Occupational Epidemiology Unit, ISPO Cancer Research and Prevention Institute, Florence, Italy; 27 Cancer Control Research, British Columbia Cancer Agency, Vancouver, British Columbia, Canada; 28 Department of Environmental Health Sciences, Yale University School of Public Health, New Haven, Connecticut, USA; 29 Sydney School of Public Health, University of Sydney, Sydney, New South Wales, Australia

## Abstract

**Background:**

Various occupations have been associated with an elevated risk of non-Hodgkin lymphoma (NHL), but results have been inconsistent across studies.

**Objectives:**

We investigated occupational risk of NHL and of four common NHL subtypes with particular focus on occupations of a priori interest.

**Methods:**

We conducted a pooled analysis of 10,046 cases and 12,025 controls from 10 NHL studies participating in the InterLymph Consortium. We harmonized the occupational coding using the 1968 International Standard Classification of Occupations (ISCO-1968) and grouped occupations previously associated with NHL into 25 a priori groups. Odds ratios (ORs) adjusted for center, age, and sex were determined for NHL overall and for the following four subtypes: diffuse large B-cell lymphoma (DLBCL), follicular lymphoma (FL), chronic lymphocytic leukemia/small lymphocytic lymphoma (CLL/SLL), and peripheral T-cell lymphoma (PTCL).

**Results:**

We confirmed previously reported positive associations between NHL and farming occupations [field crop/vegetable farm workers OR = 1.26; 95% confidence interval (CI): 1.05, 1.51; general farm workers OR = 1.19; 95% CI: 1.03, 1.37]; we also confirmed associations of NHL with specific occupations such as women’s hairdressers (OR = 1.34; 95% CI: 1.02, 1.74), charworkers/cleaners (OR = 1.17; 95% CI: 1.01, 1.36), spray-painters (OR = 2.07; 95% CI: 1.30, 3.29), electrical wiremen (OR = 1.24; 95% CI: 1.00, 1.54), and carpenters (OR = 1.42; 95% CI: 1.04, 1.93). We observed subtype-specific associations for DLBCL and CLL/SLL in women’s hairdressers and for DLBCL and PTCL in textile workers.

**Conclusions:**

Our pooled analysis of 10 international studies adds to evidence suggesting that farming, hairdressing, and textile industry–related exposures may contribute to NHL risk. Associations with women’s hairdresser and textile occupations may be specific for certain NHL subtypes.

**Citation:**

‘t Mannetje A, De Roos AJ, Boffetta P, Vermeulen R, Benke G, Fritschi L, Brennan P, Foretova L, Maynadié M, Becker N, Nieters A, Staines A, Campagna M, Chiu B, Clavel J, de Sanjose S, Hartge P, Holly EA, Bracci P, Linet MS, Monnereau A, Orsi L, Purdue MP, Rothman N, Lan Q, Kane E, Seniori Costantini A, Miligi L, Spinelli JJ, Zheng T, Cocco P, Kricker A. 2016. Occupation and risk of non-Hodgkin lymphoma and its subtypes: a pooled analysis from the InterLymph Consortium. Environ Health Perspect 124:396–405; http://dx.doi.org/10.1289/ehp.1409294

## Introduction

Non-Hodgkin lymphoma (NHL) comprises a group of malignancies that are common in industrialized countries. Studies of occupational risk factors have proven valuable for generating hypotheses regarding the possible environmental causes of NHL, and over the past four decades, these studies have produced a number of strong leads (Schottenfeld and Fraumeni 2006). In particular, occupations involving exposure to pesticides and solvents have been repeatedly associated with NHL. Other occupational risk factors have been hypothesized; these include infectious agents, sunlight, organic dusts (including flour dust, textile dust, and wood dust), mineral dusts, metals, and ionizing radiation. Nevertheless, even repeatedly observed associations (e.g., employment as farmer) have not been entirely consistent across studies. A well-defined set of occupations and potential exposures relevant to NHL etiology has yet to be established.

Among the potential reasons for the lack of consistency in previous findings is the idea that individual case–control studies lack the power to provide stable estimates of relative risk for less-common occupations and are susceptible to chance findings because of the large number of occupations evaluated. Studies differ somewhat in how occupational details are recorded, coded, analyzed, and reported, making comparison difficult, and they may not be comparable in terms of the NHL subtypes included and tumor classifications used. Finally, there may be true differences in risk associated with the same occupation across different study regions owing to local differences in population characteristics, exposure patterns, and NHL subtype distribution.

To determine the extent of agreement with previous findings in the large pooled dataset of InterLymph consortium studies, we conducted an analysis of occupations in relation to NHL using a uniform classification of occupations and NHL pathology. Our aims were *a*) to confirm the relationship of occupations of *a priori* interest to NHL and its subtypes, and *b*) to estimate the contribution of specific occupations of *a priori* interest to the incidence of NHL and its subtypes.

## Methods

*Study population.* Included in our analyses were 10 NHL case–control studies that participate in the InterLymph consortium, had collected information on occupation from cases and controls, and were willing to contribute their data to the pooled analysis (see Table 1 for the acronyms used to refer to each study, details about study designs and locations, and citations to general references for each study). The InterLymph consortium of international investigators undertakes research projects to pool data across studies that explore the etiology of lymphoid malignancies. The set of harmonized core variables, including age, sex, study center (region), smoking status, and NHL subtype, was directly obtained from the InterLymph data coordinating center. Variables on occupational history were obtained from the principal investigators of each participating study. We applied the lymphoma classification scheme for epidemiologic research developed by InterLymph investigators (Morton et al. 2007) to all participating InterLymph studies. All cases classified as “lymphoid neoplasms” according to this classification, except multiple myeloma and Hodgkin lymphoma, were included in this analysis.

**Table 1:** Description of the study population.

Study acronym	Country (study center)	Year of diagnosis	Age range (years)	Cases (*n*)	Controls (*n*)	Source of controls	Reference^*a*^
BC^*b*^	Canada (Vancouver; Victoria)	2000–2004	20–80	821	848	Random selection from client registry of the Ministry of Health; frequency matched by age, sex, and region	Spinelli et al. 2007
Nebraska^*b*^	USA (Nebraska)	1999–2002	20–75	386	533	Random digit dialing; frequency matched by age and sex	Chiu et al. 2007
NCI-SEER	USA (Detroit, MI; Iowa; Los Angeles, CA; Seattle, WA)	1998–2001	20–74	1,321	1,057	< 65 years, random digit dialing; ≥ 65 years, random selection from Centers for Medicare and Medicaid Services; stratified by study area, age, sex, and race	De Roos et al. 2005
UCSF1	USA (San Francisco, CA)	1988–1995	21–74	1,260	2,094	Random digit dialing; frequency matched by age, sex, and county of residence	Tranah et al. 2009
Yale	USA (Connecticut)	1995–2001	23–85	600	717	Women only; < 65 years, random digit dialing; ≥ 65 years, random selection from Centers for Medicare and Medicaid Services; frequency matched ± 5 years of age	Zhang et al. 2004
UK	UK (Lancashire/South Lakeland; Yorkshire; parts of southwest England)	1998–2003	18–69	827	1,129	Individually matched by age, sex, and region of residence from general practice lists	Willett et al. 2004
Epilymph	Spain; France; Germany; Italy; Ireland; Czech Republic	1998–2004	18–89	1,660	2,460	Spain/France/Ireland/Czech Republic: hospital controls matched by age (± 5 years), sex, and study region. Germany/Italy: random selection from population register; individually matched by sex, age, and study region	Cocco et al. 2010
Italy	Italy (12 areas)	1991–1993	19–79	1,910	1,771	Random sample of the population resident in the area; stratified by 5-year age groups and sex. Fortli/Ragusa/Firenze: computerized demographics files. Other areas: National Health Service files	Seniori Costantini et al. 2001
ENGELA	France (Bordeaux; Brest; Caen; Nantes; Lille; Toulouse)	2000–2004	20–75	567	722	Hospital controls, mainly in orthopedic and rheumatological departments and residing in the hospital’s catchment area; individually matched with the cases by center, age (± 3 years), and sex	Orsi et al. 2009
NSW	Australia (New South Wales; Australian Capital Territory)	2000–2001	20–74	694	694	Random selection from electoral register; frequency matched by age, sex, and state or territory	Fritschi et al. 2005
Total				10,046	12,025
Men				5,265	6,228
Women				4,781	5,797
Abbreviations: BC, British Columbia; ENGELA, l‘Etude des Facteurs Environmentaux et Genétique des Lymphomes de l’Adulte; NCI-SEER, National Cancer Institute–Surveillance, Epidemiology, and End Results Program; NSW, New South Wales; UCSF, University of California, San Francisco; UK, United Kingdom. ^***a***^One reference is given for each study, usually the publication with study results for occupation or occupational exposures. ^***b***^Only the longest-held occupation was recorded for BC and Nebraska. All other studies recorded a complete occupational history.							

*Occupational history.* For the purpose of our pooled analyses, the data on occupation were classified into a standard internationally recognized occupational classification scheme, the International Standard Classification of Occupations 1968 (ISCO-68) [International Labour Office (ILO) 1981]. Depending on the original occupational classification used by the individual studies and on whether a full-text description of the occupation was available, the ISCO-68 code for each job recorded was determined by one of the following methods: *a*) a direct conversion of the original classification to the ISCO-68 classification (for the Yale and UCSF1 studies); *b*) a direct conversion from the original classification to the ISCO-68 classification followed by checking the correctness of each ISCO-68 code by comparing it with the free-text information on the occupation (for the NCI-SEER study); *c*) using the free-text information on the occupation to individually assign the ISCO-68 code (for the BC, Nebraska, UK, and NSW studies); or *d*) directly using the original occupational codes for those studies that used ISCO-68 as their original classification (for the Epilymph, Italy, and ENGELA studies). Eight of the 10 studies collected the full occupational history of cases and controls including all occupations held for at least 1 year and starting and ending years, and 2 studies (Nebraska, BC) recorded only the longest-held occupation.

We defined occupational groups of *a priori* interest for NHL based on the peer-reviewed literature (Table 2). After discussions among three of the authors (A.’tM., A.J.D., R.V.), 25 occupational groups were constructed that included jobs associated with NHL in previous studies other than the 10 case–control studies included in our pooled analysis.

**Table 2:** Occupational groups of *a priori *interest.

Occupational group (study references reporting an increased lymphoid cancer risk)	ISCO-68 codes included in the group	Exposures hypothesized to be related to increased risk
Bakers/millers
(Alavanja et al. 1990; Blair et al. 1993)	771, Grain millers and related workers; 776, bakers, pastry cooks and confectionery makers (excludes 77650, chocolate maker and 77660, confectionery maker)	Flour dust, pesticides
Chemical workers
(Figgs et al. 1995; Franceschi et al. 1989; Ji and Hemminki 2006; Li et al. 1969; Neasham et al. 2011; Olin and Ahlbom 1980; Ott et al. 1989; Rinsky et al. 1988; Rosenman and Reilly 2004)	011, Chemists; 02510, chemical engineer (general); 02590, other chemical engineers; 03610, chemical engineering technician (general); 70040, supervisor and general foreman, chemical and related materials processing; 74, chemical processers and related workers (excludes 745, petroleum-refining workers)	Range of chemicals, benzidine, dyes
Cleaners
(Blair et al. 1993; Mester et al. 2006; ‘t Mannetje et al. 2008)	55, Building caretakers, charworkers, cleaners and related workers; 95975, building exterior cleaner	Cleaning products
Drivers
(Band et al. 2004; Cano and Pollán 2001; Holly and Lele 1997; Linet et al. 1993; ‘t Mannetje et al. 2008)	974, Earth-moving and related machinery operators (excludes 97470, concrete-mixer operator and 97475, concrete-mixing-plant operator); 979, material handling equipment operators n.e.c.^*a*^; 983, railway engine drivers and firemen; 985, motor-vehicle drivers	Engine exhausts, solvents
Dry-cleaners & laundry
(Blair et al. 1993; Cano and Pollán 2001; Ji and Hemminki 2006; Lynge et al. 2006; Schenk et al. 2009)	56, Launderers, dry-cleaners and pressers	Solvents (e.g., tetrachloroethylene)
Electrical & electronics workers
(Band et al. 2004; Figgs et al. 1995; Linet et al. 1993; Mester et al. 2006; Villeneuve et al. 2000)	023, Electrical and electronics engineers; 034, electrical and electronics engineering technicians; 70055, supervisor and general foreman, manufacturing and installation of electrical and electronic equipment; 85, electrical fitters and related electrical and electronics workers	Electromagnetic fields (EMF), solvents, polychlorinated biphenyls (PCBs)
Engine mechanics
(Blair et al. 1998; Dryver et al. 2004; Figgs et al. 1995; Hunting et al. 1995; Neasham et al. 2011; Zheng et al. 2002)	03520, Mechanical engineering technician (motors and engines); 843, motor-vehicle mechanics; 844, aircraft engine mechanics	Solvents (in particular, gasoline containing benzene)
Farmers
Meta-analyses: (Blair et al. 1992; Keller-Byrne et al. 1995, 1997; Khuder et al. 1998)	60, Farm managers and supervisors; 61, farmers; 62, agricultural and animal husbandry workers	Pesticides, infectious agents from farm animals, engine exhausts, solvents, paints, welding fumes
Farmers–animal	61240, Livestock farmer; 61250, dairy farmer; 61260, poultry farmer; 624, livestock workers; 625, dairy farm workers; 626, poultry farm workers	
Farmers–crop	61220, Field crop farmer; 61230, orchard, vineyard, and related tree and shrub crop farmer; 61270, horticultural farmer; 622, field crop and vegetable farm workers; 623, orchard, vineyard, and related tree and shrub crop workers; 627, nursery workers and gardeners; 62940, tree tapper (except rubber)	
Farmers–mixed/unspecified	60020, Farm manager; 60030, farm supervisor; 611, general farmers; 61290, other specialized farmers; 621, general farm workers; 628, farm machinery operators; 62920, apiary worker; 62930, sericulture worker; 62950, irrigator; 62960, groundsman; 62990, other agricultural and animal husbandry workers	
Fire fighters
(Band et al. 2004; Figgs et al. 1995; Ma et al. 1998; Sama et al. 1990)	581, Fire fighters	Combustion products, benzene, dioxins, chemical releases
Forestry workers
(Band et al. 2004; Blair et al. 1993; Reif et al. 1989; Woods et al. 1987; Zheng et al. 2002)	63, Forestry workers	Pesticides (herbicides), engine exhausts
Hairdressers
(Blair et al. 1993; Boffetta et al. 1994; Miligi et al. 1999; Persson et al. 1989; Seniori Costantini et al. 1998)	57, Hairdressers, barbers, beauticians, and related workers	Hair dyes, formaldehyde, solvents, ammonia
Leather workers
(Fu et al. 1996; Ji and Hemminki 2006; Linet et al. 1993; Mester et al. 2006; Neasham et al. 2011; Schenk et al. 2009)	76, Tanners, fellmongers, and pelt dressers; 79460, leather garment cutter; 79480, leather glove cutter; 79530, leather garment hand sewer; 80, shoemakers and leather goods makers	Solvents, tannins, formaldehyde, chromium
Meat workers
(McLean et al. 2004; Metayer et al. 1998; Neasham et al. 2011; Pearce et al. 1987; Tatham et al. 1997)	773, Butchers and meat preparers; 77460, meat and fish smoker	Infectious agents
Medical workers
(Eriksson et al. 1992; Figgs et al. 1995; Ji and Hemminki 2006; Lahti et al. 2008; Mester et al. 2006; Miligi et al. 1999; Schenk et al. 2009; Skov and Lynge 1991)	05260, Medical pathologist; 05430, medical science technician; 06/07, medical, dental, veterinary, and related workers	Solvents, antineoplastic drugs, night shifts, ionizing and nonionizing radiation, sterilizing agents, infectious agents
Metal processers
(Band et al. 2004; Cano and Pollán 2001; Mester et al. 2006)	70030, Supervisor and general foreman, metal processing; 72, metal processers	Metals, metal fumes
Metal workers
(Blair et al. 1993; Cano and Pollán 2001; Seniori Costantini et al. 2001; Skov and Lynge 1991; ‘t Mannetje et al. 2008; Zheng et al. 2002)	70050, Supervisor and general foreman, manufacturing of machinery and metal products; 83, blacksmiths, toolmakers, and machine tool operators; 84135, metalworking machine-tool fitter-assembler; 873, sheet-metal workers; 874, structural metal preparers and erectors	Solvents; metals; cutting, lubricating, and mineral oils
Painters
(Band et al. 2004; Dryver et al. 2004; Persson and Fredrikson 1999; Scherr et al. 1992; Schumacher and Delzell 1988; ‘t Mannetje et al. 2008)	16130, Painter, artist; 16160, painter restorer; 895, glass ceramics painters and decorators; 93, painters	Paint, solvents, paint strippers, dusts
Petroleum workers
(Franceschi et al. 1989; Thomas et al. 1982; Wong et al. 1986)	02520, Chemical engineer (petroleum); 02740, petroleum and natural gas engineer; 03620, chemical engineering technician (petroleum); 03820, petroleum and natural gas extraction technician; 713, well drillers, borers, and related workers (excludes 71380, well driller and borer except oil and gas wells and 71390, other well drillers, borers, and related workers); 74350, crude oil treater (oilfield); 745, petroleum-refining workers	Solvents, in particular benzene; gasoline
Printers
(Band et al. 2004; Blair et al. 1993; Boffetta and de Vocht 2007; Dryver et al. 2004; Rafnsson 2001; Zheng et al. 2002)	03280, Lithographic artist; 84145, printing machinery fitter-assembler; 84940, printing machinery mechanic; 92, printers and related workers	Solvents, inks, lead
Pulp & paper workers
(Band et al. 2004; Neasham et al. 2011)	733, Paper pulp preparers; 734, paper makers	Dioxins
Teachers
(Baker et al. 1999; Bernstein et al. 2002; Boffetta and de Vocht 2007; Chia et al. 2012; Dryver et al. 2004; Figgs et al. 1995; Linet et al. 1993; Miligi et al. 1999; Zheng et al. 2002)	13, Teachers	Infectious agents
Textile workers
(Blair et al. 1993; Cano and Pollán 2001; Delzell and Grufferman 1983; Fritschi and Siemiatycki 1996; Miligi et al. 1999; Schumacher and Delzell 1988)	70070, Supervisor and general foreman, production of textiles and clothing manufacturing; 75, spinners, weavers, knitters, dyers, and related workers; 79, tailors, dressmakers, sewers, upholsterers, and related workers (excludes 79460, leather garment cutter and 79480, leather glove cutter); 84150, textile machinery fitter-assembler; 84945, textile machinery mechanic	Solvents, dyes, electromagnetic fields, formaldehyde
Undertakers
(Blair et al. 1993; Hayes et al. 1990; Linos et al. 1990)	592, Undertakers and embalmers	Formaldehyde
Welders
(Band et al. 2004; Dryver et al. 2004; Fabbro-Peray et al. 2001; Persson et al. 1993; Seniori Costantini et al. 1998; Zheng et al. 2002)	872, Welders and flame-cutters	Solvents, welding fumes, metal fumes, electromagnetic fields
Wood workers
(Band et al. 2004; Boffetta and de Vocht 2007; Eriksson et al. 1992; Gallagher et al. 1985; Linet et al. 1993; Mao et al. 2000; Miller et al. 1989; Persson and Fredrikson 1999; Persson et al. 1989)	71160, Underground timberman; 731, wood treaters; 732, sawyers, plywood makers, and related wood-processing workers; 81, cabinetmakers and related woodworkers; 954, carpenters, joiners, and parquetry workers	Wood dust, solvents
^***a***^Not elsewhere classified.		

We also studied occupations within a group separately up to the detail of the 5-digit ISCO-68 code to explore whether an association was restricted to specific occupations within the group. For example, crop farmers were studied as a group, and specific occupations within this group such as orchard farmers and rice farmers were also studied separately.

*Statistical analyses.* Unconditional logistic regression was used to calculate odds ratios (ORs) and 95% confidence intervals (CIs) for the association between NHL and occupations in the pooled data set in models adjusted for age, sex, and study center. For each *a priori* occupational group and individual ISCO-68 occupation defined by a 1-digit, 2-digit, 3-digit, and 5-digit code, a dichotomous variable was created for ever having worked in that occupation. Duration of employment was coded as < 1 year, 1–10 years, and > 10 years in the occupation. Smoking status (never/former/current) was considered as a potential confounder, but adjusting for smoking made no substantial difference to the relative risk estimates (data not shown); consequently, smoking was not included as a covariate.

Analyses were performed for all NHL combined (excluding Hodgkin lymphoma and multiple myeloma) and separately for each of four major NHL subtypes [diffuse large B-cell lymphoma (DLBCL), follicular lymphoma (FL), chronic lymphocytic leukemia/small lymphocytic lymphoma (CLL/SLL), and peripheral T-cell lymphoma (PTCL)]; the same set of controls that was used for all NHL combined was used for each subtype. Two studies did not include CLL/SLL (UCSF1; UK) and were excluded from all CLL/SLL–specific analyses. All analyses were repeated stratified by sex. All statistical tests were two-sided with a significance level of 0.05. The Nebraska and BC studies included longest-held occupation only and were excluded from analyses of duration but were included in analyses of ever employment because their exclusion made little difference to the results.

Polytomous regression was used to test whether differences in ORs by NHL subtype were statistically significant at *p* < 0.05; we tested for heterogeneity in effect across the four subtypes (DLBCL, FL, CLL/SLL, PTCL) based on data for ever employment in the occupation with both sexes combined. We tested for heterogeneity among studies using Cochran’s chi-squared test or the *Q*-test (Higgins and Thompson 2002); there was no evidence of significant heterogeneity (data not shown). To identify those associations with the largest potential impact on NHL incidence under the assumption of causality and in the absence of confounding, we calculated a population attributable fraction (AF) for occupations in which 1% or more of cases had ever worked and that were associated with an increased relative risk. The formula for AF calculation used the prevalence of ever employment in each occupation in controls as an estimate of population prevalence: prevalence_controls_ × (OR – 1)/[1 + prevalence_controls_(OR – 1)] (Last et al. 1995).

*Criteria for presentation of results.* The present analysis involved many specific occupations within the 25 *a priori* groups for which previous research demonstrated an association with an increased relative risk of NHL: 925 of > 2,000 relevant codes in the ISCO-68 classification were involved in this analysis. We set criteria to determine which associations to include in the results. We present results for ever employment and for > 10 years employment for all NHL and each of the four subtypes for each occupational group of *a priori* interest regardless of whether the estimates were statistically significant, with the exception of occupational groups with < 10 cases or < 10 controls. One occupational group in the analyses of all NHL (undertakers) and two groups in the analyses of the four subtypes (pulp & paper workers and petroleum workers) were excluded from the results because they had < 10 cases or < 10 controls. Additionally, we report associations with specific occupational titles included within the occupational groups of interest if we estimated a statistically significant OR (> 1.10 or < 0.90, for ever employment or > 10 years employment) based on men and women combined for all NHL or for any one of the four subtypes.

ORs were also calculated for the 1,286 occupations that were not included in the 25 groups of *a priori* interest. These results are not presented here but are available upon request.

## Results

The 10 case–control studies included 10,046 cases and 12,025 controls (Table 1). Of the cases, 50% were from Europe, 43% were from North America, and 7% were from Australia. The year of diagnosis ranged from 1988 to 2004, and 52.4% of cases were male. The mean ± standard deviation (SD) age at interview was 57.6 ± 12.8 years for cases and 55.4 ± 14.2 years for controls. The mean year of first employment (in the 8 studies with full occupational history) was 1959 (± 16 years; range, 1915–2003) for cases and 1961 (± 16 years; range, 1912–2002) for controls. Of the four subtypes selected for separate analyses, DLBCL formed the largest group with 3,061 cases (52.4% male), followed by FL (2,140 cases; 45.6% male), CLL/SLL (1,014 cases; 59.3% male), and PTCL (632 cases; 56.5% male).

None of the 24 broad occupational groups of *a priori* interest (see Table 2) had a statistically significant positive association with NHL for ever employment (Table 3). However, one or more specific titles within 10 of these 24 groups were positively associated with NHL. There were positive associations with ever employment in cleaning occupations for “charworkers, cleaners and related” (OR = 1.17; 95% CI: 1.01, 1.36) and in electrical and electronic occupations for “electrical wiremen” (OR = 1.24; 95% CI: 1.00, 1.54); there were also positive associations with > 10 years employment for “electrical fitters & related electrical/electronics workers” and selected subgroups of these occupations. Among farming occupations, ever employment as “field crop and vegetable farm workers” (OR = 1.26; 95% CI: 1.05, 1.51) and as “general farm workers” (OR = 1.19; 95% CI: 1.03, 1.37) had a positive association with NHL. Employment of > 10 years as a forestry worker was also associated with NHL (OR = 2.25; 95% CI: 1.18, 4.32; 28 cases, 14 controls). Other positive associations were observed for NHL for ever employment as a “women’s hairdresser” (OR = 1.34; 95% CI: 1.02, 1.74), among painters as “spray-painters (except construction)” (OR = 2.07; 95% CI: 1.30, 3.29), among textile workers as “milliners and hatmakers” (OR = 2.46; 95% CI: 1.28, 4.74), and among woodworker occupations as “general carpenter” (OR = 1.42; 95% CI: 1.04, 1.93). Furthermore, > 10 years employment was positively associated with NHL among medical workers for “medical doctors” (OR = 1.87; 95% CI: 1.23, 2.85; 57 cases, 38 controls) and among metal workers for “machine-tool operators” (OR = 1.55; 95% CI: 1.11, 2.17; 84 cases; 63 controls). The occupational group of teachers was negatively associated with NHL (OR = 0.89; 95% CI: 0.81, 0.98), as were some of the specific occupations within the teachers group. Only “head teachers” had a positive association with NHL (OR = 2.16; 95% CI: 1.15, 4.06).

**Table 3:** Adjusted ORs (95% CIs) for NHL by occupational title in 24 occupational groups.

Occupational group^*a*^ and code: occupational title^*b*^	Cases (*n*)	Controls (*n*)	All NHL (*n* = 10,046)					
			Ever employed			> 10 years employment		
			Male and female	Male	Female	Male and female	Male	Female
			OR^*c*^ (95% CI)	OR^*c*^ (95% CI)	OR^*c*^ (95% CI)	OR^*c*^ (95% CI)	OR^*c*^ (95% CI)	OR^*c*^ (95% CI)
Bakers/millers	131	158	0.95 (0.75, 1.20)	1.00 (0.75, 1.35)	0.83 (0.55, 1.24)	0.90 (0.60, 1.33)	0.95 (0.60, 1.49)	—
Chemical workers	127	167	0.96 (0.76, 1.21)	0.98 (0.76, 1.28)	0.93 (0.53, 1.61)	1.00 (0.68, 1.47)	1.06 (0.70, 1.59)	—
Cleaners	534	589	1.11 (0.98, 1.25)	1.13 (0.92, 1.38)	1.12 (0.95, 1.31)	1.03 (0.84, 1.27)	1.24 (0.85, 1.82)	0.99 (0.77, 1.27)
552: Charworkers, cleaners and related	377	395	1.17 (1.01, 1.36)	1.26 (0.91, 1.75)	1.15 (0.97, 1.36)	1.05 (0.83, 1.34)	1.37 (0.75, 2.49)	1.02 (0.79, 1.33)
Drivers	787	900	1.03 (0.93, 1.14)	1.06 (0.95, 1.19)	0.77 (0.53, 1.13)	1.00 (0.85, 1.17)	1.03 (0.87, 1.21)	—
983: Railway-engine drivers and firemen	10	27	0.45 (0.22, 0.94)	0.45 (0.22, 0.94)	NA	—	—	—
98590: Other motor-vehicle drivers	56	98	0.65 (0.46, 0.92)	0.72 (0.50, 1.02)	—	0.53 (0.29, 0.96)	0.57 (0.31, 1.06)	—
Dry-cleaners	97	125	0.92 (0.70, 1.20)	0.97 (0.56, 1.69)	0.90 (0.66, 1.23)	1.29 (0.74, 2.23)	—	1.36 (0.72, 2.55)
Electrical & electronic	632	749	1.05 (0.94, 1.18)	1.08 (0.95, 1.23)	1.01 (0.79, 1.29)	1.12 (0.95, 1.32)	1.09 (0.92, 1.30)	1.47 (0.90, 2.41)
85: Electrical fitters and related electrical/electronics workers	525	589	1.10 (0.98, 1.25)	1.15 (0.99, 1.32)	1.01 (0.78, 1.30)	1.24 (1.02, 1.50)	1.18 (0.96, 1.45)	1.66 (0.99, 2.77)
853: Electrical and electronic equipment assemblers	121	145	1.07 (0.84, 1.37)	0.98 (0.61, 1.57)	1.08 (0.81, 1.44)	1.86 (1.10, 3.12)	—	1.88 (1.05, 3.36)
85390: Other electrical and electronic equipment assemblers	79	91	1.15 (0.84, 1.57)	—	1.25 (0.89, 1.76)	2.18 (1.09, 4.38)	NA	2.28 (1.12, 4.64)
855: Electrical wiremen	177	178	1.24 (1.00, 1.54)	1.28 (1.03, 1.58)	—	1.15 (0.83, 1.59)	1.14 (0.82, 1.58)	—
85540: Vehicle electrician	20	10	2.58 (1.20, 5.55)	2.60 (1.20, 5.59)	NA	—	—	NA
Engine mechanics	303	382	0.99 (0.84, 1.16)	1.00 (0.85, 1.17)	—	1.06 (0.77, 1.46)	1.08 (0.79, 1.49)	NA
Farmers–any	1,372	1,433	1.03 (0.95, 1.13)	1.02 (0.92, 1.14)	1.03 (0.89, 1.19)	1.06 (0.95, 1.19)	1.04 (0.90, 1.21)	1.06 (0.88-1.29)
Farmers–animal	264	316	0.86 (0.72, 1.02)	0.82 (0.66, 1.00)	0.90 (0.65, 1.23)	0.92 (0.72, 1.18)	0.84 (0.62, 1.14)	1.03 (0.66, 1.60)
Farmers–crop	582	573	1.10 (0.97, 1.24)	1.12 (0.96, 1.31)	1.04 (0.84, 1.28)	1.18 (1.00, 1.41)	1.25 (1.00, 1.57)	1.05 (0.79, 1.38)
622: Field crop and vegetable farm workers	276	233	1.26 (1.05, 1.51)	1.21 (0.95, 1.56)	1.29 (0.98, 1.69)	1.25 (0.96, 1.62)	1.18 (0.83, 1.67)	1.33 (0.90, 1.95)
62210: Field crop farm worker (general)	149	118	1.38 (1.07, 1.77)	1.32 (0.95, 1.83)	1.42 (0.95, 2.12)	1.29 (0.91, 1.82)	1.23 (0.80, 1.90)	1.34 (0.75, 2.40)
Farmers–mix and unspecified	716	698	1.07 (0.95, 1.20)	1.03 (0.90, 1.19)	1.13 (0.92, 1.38)	1.08 (0.93, 1.26)	1.00 (0.82, 1.21)	1.23 (0.94, 1.61)
621: General farm workers	437	404	1.19 (1.03, 1.37)	1.22 (1.02, 1.46)	1.12 (0.87, 1.43)	1.19 (0.95, 1.50)	1.07 (0.79, 1.46)	1.36 (0.95, 1.95)
Fire fighters	49	79	0.76 (0.53, 1.09)	0.72 (0.49, 1.04)	NA	0.50 (0.27, 0.93)	0.50 (0.27, 0.92)	NA
Forestry workers	66	71	1.05 (0.75, 1.48)	1.06 (0.74, 1.52)	—	2.25 (1.18, 4.32)	2.40 (1.23, 4.69)	NA
Hairdressers	154	158	1.21 (0.96, 1.52)	0.89 (0.55, 1.44)	1.28 (0.99, 1.65)	1.27 (0.88, 1.82)	1.20 (0.59, 2.45)	1.26 (0.83, 1.92)
57020: Women’s hairdresser	115	113	1.34 (1.02, 1.74)	—	1.43 (1.08, 1.89)	1.30 (0.84, 2.01)	—	1.39 (0.88, 2.19)
Leather workers	132	156	0.93 (0.73, 1.18)	0.90 (0.64, 1.26)	0.97 (0.70, 1.36)	0.87 (0.58, 1.30)	0.76 (0.44, 1.32)	1.04 (0.57, 1.87)
Meat workers	102	108	1.08 (0.81, 1.42)	1.22 (0.89, 1.68)	0.74 (0.41, 1.33)	1.09 (0.70, 1.68)	1.16 (0.70, 1.90)	—
Medical workers	681	895	0.96 (0.86, 1.07)	1.08 (0.88, 1.32)	0.91 (0.80, 1.03)	1.11 (0.96, 1.29)	1.32 (0.99, 1.77)	1.03 (0.87, 1.23)
061: Medical doctors	77	82	1.16 (0.84, 1.60)	1.06 (0.73, 1.55)	1.55 (0.83, 2.90)	1.87 (1.23, 2.85)	1.73 (1.07, 2.80)	—
062: Medical assistants	58	112	0.69 (0.50, 0.95)	0.69 (0.39, 1.24)	0.69 (0.46, 1.02)	0.85 (0.38, 1.87)	—	0.90 (0.38, 2.11)
Metal processers	133	132	1.13 (0.88, 1.45)	1.18 (0.90, 1.54)	0.84 (0.41, 1.71)	0.92 (0.60, 1.40)	0.90 (0.58, 1.40)	—
Metal workers	616	732	0.99 (0.88, 1.11)	1.04 (0.91, 1.17)	0.78 (0.57, 1.05)	0.96 (0.80, 1.15)	0.97 (0.80, 1.17)	0.84 (0.47, 1.49)
83220: Tool and die maker	34	46	0.81 (0.52, 1.28)	0.83 (0.52, 1.31)	—	0.48 (0.23, 1.00)	0.50 (0.24, 1.04)	—
834: Machine-tool operators	208	228	1.09 (0.90, 1.33)	1.13 (0.91, 1.41)	0.93 (0.58, 1.49)	1.55 (1.11, 2.17)	1.65 (1.14, 2.37)	—
Painters	206	221	1.15 (0.94, 1.39)	1.17 (0.94, 1.44)	1.06 (0.64, 1.78)	1.19 (0.87, 1.63)	1.21 (0.87, 1.68)	0.99 (0.33, 2.99)
93930: Spray-painter (except construction)	49	29	2.07 (1.30, 3.29)	2.46 (1.45, 4.15)	—	—	—	NA
Petroleum workers	12	18	0.79 (0.38, 1.67)	0.80 (0.38, 1.69)	NA	—	—	NA
Printers	175	230	0.95 (0.78, 1.17)	0.96 (0.75, 1.24)	0.95 (0.68, 1.33)	1.13 (0.80, 1.60)	1.11 (0.75, 1.63)	1.24 (0.56, 2.73)
Pulp & paper workers	16	24	0.79 (0.42, 1.50)	1.17 (0.55, 2.47)	0.24 (0.05, 1.13)	—	—	NA
Teachers	871	1,201	0.89 (0.81, 0.98)	0.88 (0.76, 1.03)	0.88 (0.78, 0.99)	0.90 (0.79, 1.03)	0.94 (0.76, 1.17)	0.87 (0.74, 1.04)
131: University and higher education teachers	189	274	0.75 (0.61, 0.90)	0.75 (0.57, 0.97)	0.76 (0.57, 1.01)	0.86 (0.65, 1.13)	0.97 (0.67, 1.40)	0.73 (0.48, 1.13)
132: Secondary education teachers	223	344	0.82 (0.69, 0.98)	0.91 (0.70, 1.18)	0.75 (0.59, 0.95)	0.81 (0.65, 1.03)	1.03 (0.73, 1.45)	0.67 (0.49, 0.92)
13940: Head teacher	29	15	2.16 (1.15, 4.06)	2.19 (1.02, 4.71)	—	—	—	—
13990: Other teachers	32	56	0.63 (0.40, 0.98)	0.49 (0.24, 1.02)	0.73 (0.42, 1.28)	—	—	—
Textile workers	728	773	1.07 (0.96, 1.20)	1.07 (0.86, 1.33)	1.08 (0.95, 1.24)	1.16 (0.98, 1.36)	1.05 (0.76, 1.43)	1.23 (1.02, 1.50)
793: Milliners and hatmakers	27	14	2.46 (1.28, 4.74)	—	—	—	—	—
Welders	174	198	1.03 (0.83, 1.27)	1.01 (0.80, 1.27)	1.06 (0.66, 1.71)	1.01 (0.69, 1.48)	0.91 (0.61, 1.36)	—
Wood workers	326	352	1.04 (0.89, 1.22)	1.04 (0.88, 1.23)	0.97 (0.58, 1.63)	1.06 (0.83, 1.36)	1.00 (0.78, 1.29)	—
95410: Carpenter, general	98	74	1.42 (1.04, 1.93)	1.40 (1.03, 1.92)	NA	1.19 (0.71, 2.00)	1.18 (0.71, 1.99)	NA
Abbreviations: —, < 10 cases or < 10 controls; NA, 0 cases or controls; ^***a***^Results are not presented for the undertakers occupational group because they included < 10 cases or < 10 controls. ^***b***^Results are presented for a specific occupational title within an occupational group if there was a statistically significantly increased or decreased risk of NHL associated with ever or > 10 years employment for men and women combined; results are excluded when there were < 10 cases or < 10 controls. ^***c***^Adjusted for age, sex, and study center.								

Table 4 presents ORs and 95% CIs for the four NHL subtypes for both sexes combined. DLBCL, the most common subtype, had positive associations with the occupational groups of hairdressers (OR = 1.47; 95% CI: 1.08, 2.00; 58 cases, 158 controls) and textile workers (OR = 1.19; 95% CI: 1.01, 1.41; 218 cases, 773 controls) as well as with specific occupations within these groups (women’s hairdresser, milliners and hatmakers, and sewers and embroiderers). Positive associations were also observed for specific occupations as “charworkers, cleaners and related workers” (OR = 1.27; 95% CI: 1.03, 1.58; 122 cases, 395 controls), “field crop & vegetable farm workers” (OR = 1.50; 95% CI: 1.15, 1.97; 79 cases, 233 controls) and its subgroup “field crop farm worker (general)” (OR = 1.48; 95% CI: 1.01, 2.17; 38 cases, 118 controls), “metal melters and reheaters” (OR = 2.31; 95% CI: 1.01, 5.26; 10 cases, 14 controls), and “special education teachers” (OR = 1.94; 95% CI: 1.01, 3.71; 14 cases, 24 controls). Forestry workers with > 10 years employment also had a positive association with DLBCL (OR = 3.04, 95% CI: 1.34, 6.90; 10 cases, 14 controls).

**Table 4:** Adjusted ORs (95% CIs) for each of four NHL subtypes by occupational title in 22 occupational groups.

Occupational group^*a*^ and code: occupational^*b*^ title	DLBCL (*n* = 3,061)		FL (*n* = 2,140)		CLL/SLL (*n* = 1,014)		PTCL (*n* = 632)		*p-*Value^*d*^
	Ever employed	> 10 years employment	Ever employed	> 10 years employment	Ever employed	> 10 years employment	Ever employed	> 10 years employment	
	OR^*c*^ (95% CI)	OR^*c*^ (95% CI)	OR^*c*^ (95% CI)	OR^*c*^ (95% CI)	OR^*c*^ (95% CI)	OR^*c*^ (95% CI)	OR^*c*^ (95% CI)	OR^*c*^ (95% CI)	
Bakers/millers	1.00 (0.70, 1.43)	0.92 (0.49, 1.73)	0.54 (0.30, 0.99)	—	0.89 (0.53, 1.48)	1.15 (0.57, 2.31)	1.53 (0.86, 2.73)	—	0.07
Chemical workers	0.85 (0.59, 1.22)	1.07 (0.60, 1.89)	1.34 (0.92, 1.97)	1.72 (0.93, 3.17)	0.52 (0.25, 1.09)	—	—	—	0.10
Cleaners	1.17 (0.98, 1.39)	1.10 (0.81, 1.50)	1.06 (0.85, 1.31)	0.97 (0.66, 1.43)	1.05 (0.77, 1.42)	1.04 (0.67, 1.61)	0.74 (0.47, 1.16)	—	0.28
552 Charworkers, cleaners, and related workers	1.27 (1.03, 1.58)	1.18 (0.83, 1.67)	1.19 (0.92, 1.53)	1.15 (0.76, 1.75)	1.14 (0.81, 1.62)	1.11 (0.68, 1.80)	0.67 (0.38, 1.18)	—	0.19
Drivers	1.00 (0.86, 1.17)	1.00 (0.79, 1.26)	1.05 (0.87, 1.27)	1.03 (0.77, 1.39)	1.02 (0.80, 1.31)	0.85 (0.60, 1.21)	0.96 (0.70, 1.32)	0.84 (0.51, 1.37)	0.96
Dry-cleaners	1.07 (0.72, 1.59)	0.92 (0.35, 2.44)	0.73 (0.42, 1.26)	—	1.21 (0.67, 2.21)	—	—	—	0.41
Electrical & electronic	0.99 (0.84, 1.17)	1.03 (0.81, 1.32)	1.10 (0.91, 1.34)	1.06 (0.79, 1.44)	1.12 (0.83, 1.50)	1.27 (0.85, 1.90)	1.04 (0.74, 1.46)	1.39 (0.88, 2.19)	0.85
851 Electrical fitters	0.76 (0.47, 1.24)	0.99 (0.50, 1.94)	1.00 (0.58, 1.73)	—	—	—	2.02 (1.03, 3.97)	—	0.11
Engine mechanics	1.12 (0.90, 1.39)	1.40 (0.92, 2.14)	0.99 (0.75, 1.31)	0.80 (0.41, 1.56)	0.89 (0.59, 1.36)	—	0.70 (0.40, 1.24)	—	0.36
Farmers–any	1.04 (0.91, 1.18)	0.92 (0.77, 1.11)	0.99 (0.84, 1.17)	1.07 (0.85, 1.35)	1.17 (0.98, 1.40)	1.16 (0.93, 1.45)	0.97 (0.74, 1.27)	0.96 (0.67, 1.38)	0.41
Farmers–animal	0.80 (0.61, 1.06)	1.07 (0.74, 1.55)	1.08 (0.79, 1.48)	0.94 (0.55, 1.59)	0.63 (0.42, 0.96)	0.69 (0.39, 1.21)	0.74 (0.43, 1.28)	—	0.18
Farmers–crop	1.19 (0.98, 1.43)	1.11 (0.83, 1.48)	1.07 (0.82, 1.38)	1.26 (0.85, 1.87)	1.11 (0.85, 1.43)	1.32 (0.97, 1.79)	1.09 (0.76, 1.57)	1.21 (0.75, 1.96)	0.88
622 Field crop and vegetable farm workers	1.50 (1.15, 1.97)	1.16 (0.76, 1.78)	1.10 (0.73, 1.65)	1.04 (0.53, 2.04)	1.20 (0.85, 1.69)	1.49 (0.98, 2.27)	1.35 (0.82, 2.22)	—	0.46
62210 Field crop farm worker (general)	1.48 (1.01, 2.17)	1.09 (0.60, 1.98)	1.06 (0.57, 1.95)	—	1.40 (0.91, 2.13)	1.56 (0.93, 2.60)	1.73 (0.95, 3.17)	—	0.61
Farmers–mix and unspecified	1.01 (0.84, 1.20)	0.88 (0.68, 1.14)	1.00 (0.79, 1.25)	1.07 (0.79, 1.45)	1.30 (1.06, 1.60)	1.17 (0.88, 1.55)	0.84 (0.57, 1.23)	0.95 (0.57, 1.57)	0.11
621 General farm workers	1.13 (0.90, 1.42)	1.01 (0.69, 1.49)	1.16 (0.87, 1.56)	0.91 (0.52, 1.59)	1.44 (1.13, 1.84)	1.38 (0.94, 2.03)	1.14 (0.74, 1.78)	1.27 (0.65, 2.47)	0.32
Fire fighters	0.62 (0.35, 1.13)	—	0.78 (0.41, 1.49)	—	—	—	—	—	0.86
Forestry workers	1.10 (0.66, 1.83)	3.04 (1.34, 6.90)	—	—	1.24 (0.64, 2.43)	—	—	—	0.51
Hairdressers	1.47 (1.08, 2.00)	1.51 (0.92, 2.49)	0.92 (0.60, 1.39)	—	1.79 (1.06, 3.03)	2.09 (1.01, 4.34)	—	—	0.06
57020 Women’s hairdresser	1.60 (1.13, 2.27)	1.44 (0.79, 2.62)	0.97 (0.61, 1.55)	—	2.69 (1.43, 5.06)	—	—	NA	0.03
Leather workers	0.94 (0.65, 1.37)	0.83 (0.43, 1.61)	1.10 (0.69, 1.75)	—	0.59 (0.31, 1.10)	—	1.46 (0.78, 2.76)	—	0.28
Meat workers	1.14 (0.76, 1.70)	1.53 (0.86, 2.70)	1.19 (0.75, 1.89)	—	0.85 (0.42, 1.73)	—	1.22 (0.56, 2.65)	—	0.84
Medical workers	0.85 (0.72, 0.99)	0.99 (0.79, 1.24)	1.01 (0.85, 1.19)	1.19 (0.93, 1.51)	1.10 (0.81, 1.49)	1.38 (0.95, 2.02)	1.12 (0.82, 1.52)	1.13 (0.71, 1.80)	0.17
061 Medical doctors	1.06 (0.66, 1.71)	1.46 (0.79, 2.72)	1.29 (0.77, 2.18)	2.23 (1.17, 4.26)	—	—	—	—	0.93
Metal processers	1.34 (0.95, 1.90)	1.14 (0.62, 2.08)	0.89 (0.52, 1.51)	—	1.32 (0.82, 2.12)	1.57 (0.78, 3.14)	0.92 (0.42, 1.99)	0.66 (0.16, 2.74)	0.35
723 Metal melters and reheaters	2.31 (1.01, 5.26)	—	—	NA	—	NA	—	NA	0.18
Metal workers	0.91 (0.76, 1.09)	0.86 (0.65, 1.15)	1.04 (0.84, 1.29)	1.17 (0.84, 1.64)	1.18 (0.93, 1.52)	1.14 (0.80, 1.64)	0.66 (0.45, 0.99)	0.71 (0.39, 1.29)	0.05
834 Machine-tool operators	0.92 (0.68, 1.25)	1.34 (0.81, 2.21)	1.21 (0.84, 1.75)	1.73 (0.93, 3.20)	1.08 (0.69, 1.69)	1.96 (1.04, 3.69)	0.84 (0.45, 1.58)	—	0.56
Painters	1.03 (0.77, 1.39)	1.22 (0.77, 1.94)	1.34 (0.95, 1.89)	1.27 (0.71, 2.28)	0.97 (0.61, 1.55)	1.31 (0.70, 2.46)	1.80 (1.14, 2.84)	—	0.12
93 Painters	1.06 (0.78, 1.44)	1.31 (0.81, 2.13)	1.40 (0.98, 1.99)	1.23 (0.66, 2.31)	1.05 (0.64, 1.72)	1.53 (0.77, 3.03)	1.74 (1.07, 2.83)	—	0.22
93930 Spray-painter (except construction)	1.74 (0.90, 3.37)	—	2.67 (1.36, 5.25)	—	—	—	1.31 (0.31, 5.62)	—	0.61
Printers	0.88 (0.65, 1.19)	1.36 (0.85, 2.19)	1.16 (0.83, 1.61)	1.02 (0.52, 2.01)	1.37 (0.85, 2.22)	1.27 (0.61, 2.67)	0.92 (0.50, 1.70)	—	0.38
922 Printing pressmen	—	—	—	—	6.52 (2.79-15.21)	—	—	NA	0.02
Teachers	0.88 (0.76, 1.01)	0.88 (0.72, 1.08)	0.95 (0.82, 1.11)	0.93 (0.74, 1.17)	0.99 (0.76, 1.30)	1.00 (0.69, 1.43)	0.94 (0.70, 1.26)	1.13 (0.75, 1.70)	0.79
131 University and higher education teachers	0.81 (0.61, 1.07)	0.89 (0.59, 1.33)	0.62 (0.44, 0.89)	0.72 (0.43, 1.19)	0.85 (0.49, 1.47)	—	1.03 (0.59, 1.80)	—	0.37
134 Preprimary education teachers	0.89 (0.61, 1.31)	1.46 (0.76, 2.78)	0.98 (0.66, 1.46)	—	2.00 (1.04, 3.87)	—	—	—	0.10
135 Special education teachers	1.94 (1.01, 3.71)	—	—	—	—	—	—	NA	0.54
Textile workers	1.19 (1.01, 1.41)	1.20 (0.93, 1.54)	0.94 (0.75, 1.17)	0.85 (0.59, 1.23)	1.01 (0.78, 1.30)	0.93 (0.64, 1.35)	1.60 (1.18, 2.17)	2.18 (1.45, 3.30)	0.02
75 Spinners, weavers, knitters, dyers, and related workers	1.09 (0.84, 1.42)	1.08 (0.73, 1.61)	0.81 (0.55, 1.18)	0.73 (0.39, 1.38)	1.08 (0.74, 1.56)	1.09 (0.62, 1.89)	1.85 (1.21, 2.83)	1.90 (1.00, 3.64)	0.03
79 Tailors, dressmakers, sewers, upholsterers, and related workers	1.20 (0.99, 1.47)	1.23 (0.90, 1.69)	1.02 (0.79, 1.31)	1.03 (0.67, 1.59)	0.94 (0.68, 1.30)	0.86 (0.52, 1.41)	1.35 (0.92, 1.99)	2.29 (1.38, 3.77)	0.41
793 Milliners and hatmakers	2.90 (1.30, 6.45)	—	—	—	—	NA	NA	NA	0.62
795 Sewers and embroiderers	1.51 (1.16, 1.96)	1.56 (1.00, 2.42)	1.01 (0.71, 1.44)	—	1.05 (0.68, 1.63)	1.09 (0.55, 2.19)	1.26 (0.72, 2.21)	—	0.20
Welders	1.31 (0.99, 1.74)	1.20 (0.70, 2.05)	0.81 (0.53, 1.23)	1.25 (0.63, 2.49)	0.97 (0.59, 1.60)	—	0.66 (0.31, 1.42)	1.08 (0.39, 3.02)	0.09
Wood workers	1.12 (0.89, 1.41)	1.22 (0.85, 1.75)	0.95 (0.70, 1.29)	0.97 (0.58, 1.62)	1.01 (0.71, 1.43)	0.95 (0.56, 1.60)	1.54 (1.04, 2.27)	2.04 (1.19, 3.50)	0.15
811 Cabinetmakers	0.72 (0.41, 1.28)	—	0.98 (0.51, 1.86)	—	—	—	2.41 (1.22, 4.74)	—	0.04
95410 Carpenter, general	1.14 (0.71, 1.81)	—	1.49 (0.91, 2.44)	—	2.10 (1.08, 4.09)	—	—	—	0.18
Abbreviations: —, < 10 cases or < 10 controls; NA, 0 cases or controls. ^***a***^Results are not presented for the following occupational groups because they included < 10 cases or < 10 controls: Pulp & paper workers, petroleum workers, and undertakers. ^***b***^Results are presented for a specific occupational title within an occupational group if there was a statistically significantly increased or decreased OR for at least one subtype associated with ever employment or > 10 years employment for men and women combined; results are excluded when there were < 10 cases or < 10 controls. ^***c***^Adjusted for age, sex, and study center. ^***d***^*Q*-test for heterogeneity across the four subtypes, based on ORs for ever employment in the occupation for men and women combined.									

Positive associations were present for FL with specific occupations such as “spray-painter (except construction)” (OR = 2.67; 95% CI: 1.36, 5.25; 13 cases, 29 controls) and with > 10 years employment as a “medical doctor” (OR = 2.23, 95% CI: 1.17, 4.26; 13 cases, 38 controls).

CLL/SLL was associated with ever employment in the occupational group of hairdressers (OR = 1.79; 95% CI: 1.06, 3.03; 18 cases, 130 controls), both for the specific occupation “women’s hairdresser” as well as for > 10 years employment in the occupational group of hairdressers (OR = 2.09, 95% CI: 1.01, 4.34; 10 cases, 40 controls). We observed positive associations with CLL/SLL for specific occupations such as “general farm worker” (OR = 1.44; 95% CI: 1.13, 1.84; 102 cases, 399 controls), printing pressmen (OR = 6.52; 95% CI: 2.79, 15.21; 10 cases, 19 controls), “pre-primary education teachers” (OR = 2.00; 95% CI: 1.04, 3.87; 11 cases, 111 controls) and carpenters (OR = 2.10; 95% CI: 1.08, 4.09; 13 cases, 69 controls). CLL/SLL was also associated with > 10 years employment as machine tool operators (OR = 1.96; 95% CI: 1.04, 3.69; 15 cases, 46 controls).

Three occupational groups had positive associations with PTCL: ever employment as painters (OR = 1.80; 95% CI: 1.14, 2.84; 22 cases, 221 controls), textile workers (OR = 1.60; 95% CI: 1.18, 2.17; 56 cases, 773 controls), and wood workers (OR = 1.54; 95% CI: 1.04, 2.27; 31 cases, 352 controls); the last two occupational groups also had increased ORs for > 10 years employment. Specific textile occupations associated with PTCL included “spinners, weavers, knitters, dyers and related workers” (OR = 1.85; 95% CI: 1.21, 2.83; 27 cases, 313 controls) and “tailors, dressmakers, sewers, upholsterers and related workers” (> 10 year OR = 2.29, 95% CI: 1.38, 3.77, 19 cases, 183 controls). The specific wood worker occupation associated with PTCL was “cabinet makers” (OR = 2.41; 95% CI: 1.22, 4.74; 10 cases, 81 controls). PTCL was also associated with “electrical fitters” (ever employed OR = 2.02; 95% CI: 1.03, 3.97; 10 cases, 92 controls).

Evidence of heterogeneity in relative risks (*p* < 0.05, *Q*-test for heterogeneity) across the four NHL subtypes was present for “women’s hairdressers,” metal workers, “printing pressmen,” textile workers, and “cabinetmakers” (Table 4). “Printing pressmen,” however, had very small numbers of cases and controls (< 10) for all analyses except for CLL/SLL.

*Attributable fraction.* We estimated the proportion of NHL and of each subtype that was attributable to the main occupational groups (farmers, textile workers, hairdressers, wood workers, painters) or to specific occupations (e.g., “women’s hairdressers,” “spray-painters”) for which an elevated relative risk had been observed (*p* < 0.05). AFs for NHL were low, between 0.3% for “women’s hairdressers” and 0.63% for “general farm workers,” and were somewhat higher for the rarer individual subtypes: 1.49% for “women’s hairdressers” and CLL/SLL and ≥ 3.69% for the textile worker group and PTCL. AFs differed by sex in a number of occupations, reflecting the scarcity of men or women in a particular occupation.

## Discussion

We found evidence that NHL was associated with employment as textile workers, hairdressers, and farm workers, as well as with employment as painters, printers, wood workers, metal workers, medical workers, electrical workers, and cleaners. The statistically significant heterogeneity in relative risk estimates among subtypes suggested that employment as “women’s hairdressers” was particularly associated with DLBCL and CLL/SLL and employment as textile workers with DLBCL and PTCL.

Our pooled analysis used a uniform classification of NHL diagnosis and was substantially larger than any individual study. A limitation of our study is that grouping workers according to job title disregards the wide qualitative and quantitative variation in exposure that may occur for workers with the same job title (McGuire et al. 1998). Even if an association between job title and disease is found, the potentially causative agents are unknown, although they are likely to be common rather than rare exposures within the occupational group. The international nature of this study also implies that only associations for occupations with internationally comparable exposure profiles can be detected and that some misclassification will be introduced owing to the recoding of different occupational classifications into a single one. An advantage of using job titles rather than specific exposures is that recall by participants is less likely to be influenced by their disease status, making differential misclassification also less likely. The multiple comparisons of a job title–based approach, however, suggest a vulnerability to false positive findings. Results are therefore focused on the *a priori*–selected occupational groups (24 were eligible) extracted from earlier NHL research. We discuss below the findings from our study that are consistent with previously reported associations, and we also discuss occupational exposures that might be implicated as etiologic agents.

We confirmed the previously reported association of NHL with crop farming occupations (Blair et al. 1992; Keller-Byrne et al. 1997), but not with animal farming (Amadori et al. 1995; Boffetta and de Vocht 2007; Lee et al. 2002), which was negatively associated with CLL/SLL. This finding suggests that risk estimates for all farming and all NHL combined may be uninformative and that future studies will need to consider both NHL subtype and farming type to identify the possible specific farming exposures that may be involved in these associations.

The observed associations for hairdressers were stronger for women’s hairdressers than for other job titles within this occupational category, supporting a hypothesis of hair dye or other hair treatments more commonly used by women than by men or children as possible causes. Associations were present for DLBCL and CLL/SLL but were absent for FL. A previous pooled analysis of InterLymph studies reported associations with personal hair dye use for NHL subtypes FL and CLL/SLL (Zhang et al. 2008). Exposure from personal hair dye use is, however, not strictly comparable to the exposure experienced by hairdressers because hairdressers are exposed on a daily basis to a range of other compounds such as solvents and propellant gases, including dichloromethane and chlorofluorocarbons.

The observed associations between textile-related occupations and NHL (DLBCL and PTCL) suggest a range of possible exposures that can occur in this environment, but the implication of multiple specific occupations within this group, which is involved in both fabric making and garment making, indicates that associations were not restricted to specific tasks in the textile industry (e.g., textile dyeing) but rather may be associated with more ubiquitous exposures (Siemiatycki et al. 1986).

We found associations with NHL for a number of other occupations potentially exposed to solvents. Among these occupations were cleaners, painters (especially spray-painters) with potential for exposure to solvents in paints and paint strippers, and machine tool operators, who may be exposed to a range of solvents including aliphatic hydrocarbon solvents, aromatic hydrocarbon solvents, chlorinated solvents, mineral oils, and diesel fuel and exhaust. Metal workers would also be exposed to metal dust and metal-working fluids. Although our findings of positive associations for these occupations may support a role for solvent exposure as a risk factor for NHL, other exposures may also be responsible.

Some solvent exposure would likely be implicated in two other occupational groups for which we observed an association with NHL: several specific occupations within the electrical and electronics–related group may also have exposure to electromagnetic fields (Mester et al. 2006), and carpenters may be exposed to wood dust, wood preservatives, formaldehyde, and molds in addition to solvents. Forestry workers could also be exposed to wood dust and potentially to pesticides and engine exhausts.

All teaching occupations combined were inversely associated with NHL, a finding that is the opposite of the results of a death certificate–based case–control study (Figgs et al. 1995) and a meta-analysis (Baker et al. 1999). We did observe a marked positive association for preprimary education teachers with CLL/SLL, which could point towards common childhood infections as a possible causal factor (Vineis et al. 2000). Long-term employment as a medical doctor, in which infectious agents may also play a role, was associated with FL.

Among the four NHL subtypes, the statistically significant heterogeneity in relative risk estimates suggested that “women’s hairdressers” were at an increased risk for DLBCL and CLL/SLL, but not for FL, which was previously suggested to be associated with personal hair dye use (Zhang et al. 2008; Sangrajrang et al. 2011). Textile workers were another occupational group to show heterogeneity across NHL subtypes and were particularly at risk for DLBCL and PTCL. There was no significant heterogeneity in ORs for crop and mixed/unspecified farming among the four subtypes, although DLBCL and CLL/SLL appeared to be most strongly associated with farming occupations. We note the strong association between spray-painters and FL as well as the lack of adequate numbers for analysis in the other subtypes examined. The authors of a recent major analysis of NHL subtypes and a broad range of risk factors in the InterLymph consortium reported that certain occupations were associated with one or more subtypes, including spray-painters (FL), crop farmers (DLBCL, CLL/SLL), hairdressers (DLBCL, CLL/SLL), and medical doctors (FL). These analyses were adjusted for all other significant risk factors (Morton et al. 2014) and are consistent with our findings. However, our analysis based on occupational titles is not the proper setting in which to explore whether socioeconomic confounders for which we were unable to control might have generated some of our positive findings; such hypotheses need to be specifically addressed in dedicated analyses.

## Conclusions

This pooled analysis supports a role for textile-, hairdressing-, and farming-related exposures in the development of NHL. Additional occupations associated with NHL or NHL subtypes include cleaners, painters, printers, and wood workers. The results by sex indicate that occupational exposures may play a role in NHL for both women and men, but the specific occupations involved differ between the sexes. The large numbers of participants and the application of standard NHL and occupational classification systems allowed us to make estimates of relative risk by NHL subtype, forming an important step towards improving our understanding of NHL etiology. The findings of the present study can be further refined at the next stage, after specific exposures are identified in detailed exposure studies.
